# Psoriasis and Cardiovascular Diseases: An Immune-Mediated Cross Talk?

**DOI:** 10.3389/fimmu.2022.868277

**Published:** 2022-05-24

**Authors:** Gloria Orlando, Barbara Molon, Antonella Viola, Mauro Alaibac, Roberta Angioni, Stefano Piaserico

**Affiliations:** ^1^Unit of Dermatology, Department of Medicine - DIMED, University of Padova, Padova, Italy; ^2^Department of Biomedical Sciences – DSB, University of Padova, Padova, Italy; ^3^Istituto di Ricerca Pediatrica, Città della Speranza – IRP, Padova, Italy

**Keywords:** psoriasis, cytokines, endothelial dysfunction (ED), cardiovascular diseases, inflammation

## Abstract

Psoriasis is a chronic immune-mediated inflammatory skin disease, characterized by well-demarcated scaly, erythematous, infiltrated plaques. The cutaneous-to-systemic expansion of the inflammation in psoriasis leads to the concept of “psoriatic march” or “inflammatory skin march”. Accordingly, psoriasis is thought to be a systemic inflammatory disease associated with numerous comorbidities. Indeed, it’s currently considered an independent risk factor for cardiovascular diseases. Here, we discuss the current knowledge on TNF-α and IL-23/IL-17 mediated pathways linking the psoriatic plaque to the cardiovascular compartment. We further argue the possible involvement of the endothelial compartment in the psoriatic plaque- cardiovascular system crosstalk.

## The Psoriatic March (From the Cutaneous to Systemic Spreading of the Inflammation)

Psoriasis is a chronic cutaneous disease resulting from a complex interplay between immunological, environmental and genetic factors, with a global prevalence ranging from 0.09 to 5.1% of adult population (1). It predominantly affects the skin with a broad spectrum of cutaneous manifestations that comprise either solitary lesions, varying from pinpoint to large plaques, or generalized erythroderma. 85-90% of all patients present a plaque-type psoriasis that appears with demarcated, scaly, erythematous and infiltrated lesions. These manifestations are usually located in specific body sites, as for instance the extensor surfaces of the limbs (knee and elbows) and other mechanically stressed sites (lumbar region) (2, 3). At the bottom of psoriasis clinical signs there is a complex multistep process involving a dysregulated activation of both the innate and adaptive immunity. Indeed, nowadays the prevailing model for psoriatic pathophysiology implies crucial abnormalities in the antigen presentation by dendritic cells (DCs), and the concomitant altered differentiation of T helper (T_H_) cell populations. Here, the enhanced (IL)-17-producing T cell response promotes the recruitment of immune cells, thus driving the psoriatic plaque formation (4–6).

Beyond the skin, systemic inflammation has been reported in psoriatic patients leading to the concept of “psoriatic march”, or “inflammatory skin march” (7). In accordance with this hypothesis, the inflammatory cutaneous-to-systemic expansion in psoriatic patients might cause the insurgence of systemic immune-mediated alterations, subsequently leading to important comorbidities such as diabetes mellitus, metabolic disorders, including obesity, hypertension, dyslipidemia, and cardio-vascular diseases (CVDs) (8, 9). Consistently, Sokolova et al. reported an increase of inflammatory serum markers lipocalin 2, beta-defensin 2, IL-22, IL-8 and calprotectin in psoriatic patients (9, 10). Moreover, several circulating biomarkers of inflammation, as C-reactive protein (CRP), erythrocyte sedimentation rate and the platelet activation marker P-selectin, positively correlate with the disease severity (11–13). In this line, elevated levels of pro-inflammatory S100A12, CCL22, IL1RN, CCL2, VEGF, ICAM1, IL-15, TNF, CCL5 have been identified both in the skin and in the blood of psoriatic patients, thus highlighting that psoriatic plaque pathological products might circulate toward the vascular network (14). Indeed, numerous foci of inflammation within the skin, liver, joints, tendons, and aorta have been observed in psoriasis patients by (18)F-fluorodeoxyglucose positron emission tomography computed tomography (FDG-PET/CT) (15). These results not only point out the systemic burden of psoriasis, but also suggest that psoriatic inflammation might act at distant sites.

In this review, we will overview current knowledge on the association between psoriasis and cardiovascular (CV) risk with a special focus on immune players contributing to the pathogenic link between psoriasis and CVDs.

## Psoriasis as an Independent Cardiovascular Risk Factor

A high prevalence of underdiagnosed and undertreated CV risk factors, including hypertension, diabetes, hyperlipidemia, obesity, and metabolic syndrome, is commonly reported in psoriatic patients (**Table 1**) (16–31). However, the undoubted causality of this correlation is still questioned.

**Table 1:** Summary of systemic reviews and meta-analysis analyzing the association between psoriasis and traditional cardiovascular risk factors.

References	Study population	Traditional cardiovascular risk factors and relative risk of measures
Armsrong et al., (17)	Psoriasis: 20831 Control: 1898169	Obesity OR 1.66 (1.46-1.89); mild psoriasis OR 1.46 (1.17–1.82); severe psoriasis OR 2.23 (1.63–3.05)
Armstrong et al., (16)	Psoriasis: 309469 Controls: 2088197	Hypertension: all psoriasis, OR = 1.58 (1.42–1.76); mild psoriasis, OR = 1.30 (1.15–1.47); severe psoriasis, OR = 1.49 (1.20–1.86)
Ma et al., (18)	Psoriasis: 238385 Controls: 2102220	Dyslipidemia OR (1.04-5.05)
Armstrong et al., (19)	Psoriasis: 314036 Control: 3717217	Diabetes OR 1.59 (1.38–1.83); mild psoriasis pooled OR 1.53 (1.16–2.04); severe psoriasis pooled OR 1.97 (1.48–2.62)
Armstrong et al., (20)	Psoriasis: 41853 Control: 135814	Metabolic syndrome OR 2.26 (1.70–3.01)
Coto-Segura et al., (21)	Psoriasis: 557697 Control: 5186485	Type 2 diabetes pooled OR 1.76 (1.59–1.96)
Miller at al., (22)	Psoriasis: 503686 Control: 2686694	Diabetes OR 1.9 (1.5–2.5); hypertension OR 1.8 (1.6–2.0), dyslipidemia OR 1.5 (1.4–1.7); obesity OR 1.8 (1.4–2.2); metabolic syndrome OR 1.8 (1.2–2.8)
Rodriguez-Zuniga et al., (23)	Psoriasis: 25042 Control: 131609	Metabolic syndrome pooled OR 1.42 (1.28–1.65)
Singh et al., (24)	Psoriasis: 46714 Control: 1403474	Metabolic syndrome pooled OR 2.14 (1.84–2.48)
Mamizadeh et al., (25)	Psoriasis: 922870 Control: 12808071	Diabetes OR 1.69 (1.51–1.89)
Choudhary et al., (26)	Psoriasis: 15939 Control: 103984	Metabolic syndrome OR 2.077 (1.84–2.34)
Choudhary et al., (27)	Psoriasis: 17672 Control: 66407	Increased systolic blood pressure OR 2.31 (1.12–4.74); diastolic blood pressure OR 2.31 (1.58–3.38); abdominal obesity OR 1.90 (1.45–2.50); Triglycerides OR 1.80 (1.29–2.51)
Phan et al., (28)	Pediatric psoriasis: 43808 Control: 5384057	Obesity OR 2.45 (1.73–3.48); diabetes OR 2.32 (1.34–4.03); hypertension OR 2.19 (1.62–2.95); hyperlipidemia OR 2.01 (1.66–2.42); metabolic syndrome OR 1.75 (1.75–7.14)
Duan et al., (29)	Psoriasis: 255132 Control: 814631	Hypertension OR 1.43 (1.25–1.64)
Cho et al., (30)	Pediatric psoriasis: 20676 Controls: 5239197	Obesity pooled OR 2.4 (1.6-3.59); diabetes OR 2.01 (1.09-3.73); hypertension OR 2.73 (1.79-4.17); dyslipidemia OR 1.67 (1.42-1.97); metabolic syndrome OR 7.49 (1.86-30.07)

OR, odds ratio. Values in brackets indicate 95% confidence intervals.

Obesity itself is an independent risk factor for the psoriatic development due to the massive induction of systemic inflammatory responses (17). Nevertheless, multiple studies have demonstrated an increased prevalence of obesity among patients with psoriasis. Although the molecular mechanisms have still to be elucidated, a severity-dependent relationship between the two diseases has been demonstrated (32, 33). Similar findings were found for hypertension, having psoriatic patients higher rate incidence of hypertension in comparison to the global population (34). To note, severe psoriasis patients showed a higher prevalence of hypertension compared with mild psoriasis patients (29). Several pathways have been proposed to unfold this association, as the higher serum levels of the renin and angiotensin-converting enzyme (ACE) in psoriatic patients (34). Furthermore, reduced blood levels of high-density lipoprotein (HDL) were described in psoriatic patients compared to age-matched controls (18, 35, 36). Intriguingly, moderate-to-severe psoriasis has been often associated to metabolic syndrome (MetS), which represents a well-known set of risk factors, including obesity and hypertension, but also dyslipidemia, insulin resistance related with an elevated risk of type 2 diabetes and CVD (19). Recent meta-analyses and epidemiological studies further highlighted the prevalence of MetS in 20-50% of psoriatic patients in comparison to healthy subjects (20, 24, 37, 38). In this line, Langan et al. demonstrated that psoriasis was independently associated with MetS even after adjustments for multiple parameters including age, sex, follow-up, smoking, and social class and that importantly, MetS prevalence directly correlates with the cutaneous disease extent (37). Together, these reports indicated that conventional CV risk factors are prevalent among psoriatic patients. Beside this notion, psoriasis *per se* is increasingly thought to be an independent risk factor for CVD and a “dose effect” of psoriasis’ activity on the patients’ cardiovascular risk has been demonstrated (39). Systemic reviews and meta-analysis evaluating the association between psoriasis and CVDs are summarized in **Table 2** (22, 28, 40–49).

**Table 2:** Summary of systemic reviews and meta-analysis assessing the risk of cardiovascular diseases in psoriasis.

References	Study population	CV outcomes and relative risk of measures
Xu et al., (40)	Psoriasis: 326598 Controls: 5230048	MI and stroke RR 1.2 (1.1-1.31); MI RR 1.22 (1.05-1.42; Stroke RR 1.21 (1.04-1.40)
Armstrong et al., (41)	Psoriasis: 218654 (mild 201239; severe 17415) Controls: 9914799	Mild Psoriasis: MI RR 1.29 (1.02–1.63), stroke RR 1.12 (1.08–1.16) Severe Psoriasis: MI RR 1.70 (1.32–2.18), stroke RR 1.56 (1.32–1.84), CV mortality RR 1.39 (1.11–1.74)
Gaeta et al., (42)	Psoriasis: 1862297 Controls: 43407300	Overall CV RR 1.24 (1.18–1.31), MI RR 1.24 (1.11–1.39) Vascular disease RR 1.27 (1.12–1.43), CV Mortality RR 1.41 (0.97–2.04) MI and stroke RR 1.20 (1.10–1.31)
Gu et al., (43)	Psoriasis and controls: 6230774	Stroke RR 1.26 (1.12–1.41) MI RR 1.32 (1.13–1.55) CVD RR 1.47 (1.30–1.60) CV mortality RR 1.33 (1.00–1.77)
Horreau et al., 2013 (44)	Psoriasis: 324650 Controls: 5309087	Cohort studies: MI OR 1.25 (1.03–1.52), CAD OR 1.20 (1.13–1.27), stroke OR 1.02 (0.92–1.14) Cross-sectional studies: MI OR 1.57 (1.08–2.27), CAD OR 1.84 (1.09-3.09), stroke OR 1.14 (1.08–1.19)
Miller et al., (22)	Psoriasis: 503686 Controls: 29686694	Overall CVD OR 1.4 (1.2–1.7) IHD OR 1.5 (1.2–1.9) Cerebrovascular disease OR 1.1 (0.9–1.3) CV mortality OR 0.9 (0.4–2.2)
Pietrzak et al., (45)	Psoriasis: 367358 Controls: 9199656	CV events OR 1.28 (1.18–1.38)
Samarasasekera et al., (46)	Psoriasis: 488315 (mild: 327418; severe: 12854) Controls: 10024815	All psoriasis: MI HR 1.40 (1.03–1.89), stroke HR 1.13 (1.01–1.26) Mild psoriasis: CVD mortality SMR 1.03 (0.86–1.25), MI HR 1.34 (1.07– 1.68), stroke HR 1.15 (0.98–1.35) Severe psoriasis: CVD mortality SMR 1.37 (1.17–1.60), CVD mortality HR 1.57 (1.26–1.96), MI HR 3.04 (0.65–14.35), stroke HR 1.59 (1.34–1.89)
Richard et al., (47)	NA	Cohort studies: MI OR = 1.25 (1.03-1.52); CAD1.20 (1.13-1.27); Cross-sectional studies: MI OR = 1.57 (1.08-2.27)], CAD OR = 1.19 (1.14-1.24); Case-control studies: CAD OR = 1.84 (1.09-3.09)
Raaby et al., (48)	NA	Mild psoriasis: MI HR 1.2 (1.06-1.35); stroke HR 1.10 (1.0-1.19); CV death HR 1.06 (0.90-1.24) Severe psoriasis: MI HR 1.70 (1.18-2.43); stroke HR 1.38 (1.20-1.60); CV death HR 1.37 (1.13-1.67)
Dhana et al., (49)	Psoriasis: 285675 Controls: NA	All psoriasis: CV mortality pooler RR 1.15 (1.09-1.21); mild psoriasis: CV mortality pooled RR 1.05 (0.92-1.20); severe psoriasis: CV mortality pooled RR 1.38 (1.09-1.74)
Phan et al., (28)	Pediatric psoriasis: 43808 Controls: 5384057	IHD or HF OR 3.15 (1.06-9.42)

CAD, coronary artery disease; CV, cardiovascular; HF, heart failure; HR, hazard ratio; IHD, ischemic heart disease; MACE, major adverse cardiovascular events; MI, myocardial infarction; OR, odds ratio; RR, relative risk; SMR, standardized mortality ratio. Values in brackets indicate 95% confidence intervals. NA, not applicable.

In 2006, Gelfand et al. conducted the seminal study identifying psoriasis as an independent risk factor for myocardial infarction (MI) taking advantage of prospective data from the United Kingdom General Practice Research Database (50). Here, the incidence of MI per 1000 person-years was 3.58 for control patients (95% CI 3.52-3.65), 4.04 for patients with mild psoriasis (95% CI 3.88-4.21) and 5.13 for patients with severe psoriasis (95% CI 4.22-6.17). Furthermore, after adjusting for hypertension, diabetes mellitus, and hyperlipidemia younger patients showed a greater relative risk (RR) for MI. In line with this, Samarasekera et al. and Armstrong et al. assessed the risk of CVDs considering the severity of psoriasis and noticed an increased risk of major cardiovascular events (MACE), namely myocardial infarction and stroke, and CV mortality in severe psoriasis patients (41, 46). Moreover, Egeberg et al. found that longer disease duration had a stronger association with the risk of MACE (51).

Accordingly, psoriasis affects the Framingham Risk Score for over 60% of patients being thus identified as an independent CV risk factor (52). Strengthened by this convincing evidence, psoriasis has been recently included in the European and American Guidelines on Cardiovascular disease prevention as a (1.5) risk factor multiplier for CV risk (53) and, in adults 40 to 75 years of age without diabetes mellitus and a CV intermediate risk, as a risk-enhancing factor favoring initiation of statin therapy (54). Moreover, the Joint American Academy of Dermatology- National Psoriasis Foundation Guidelines recommend that patients with psoriasis should be advised of their increased cardiovascular risk and referred to the primary care physician or cardiologist (**Table 3**) (55).

**Table 3:** Psoriasis and cardiovascular diseases comorbidity strength of recommendation and level of evidence.

Recommendation	Strength of recommendation	Level of evidence
Risk assessment Recommended for all patients with psoriasis	B	II-III
Screening Early and more frequent screening for hypertension, diabetes, and hyperlipidemia in patients who are candidates for systemic or phototherapy or who have psoriasis involving >10% body surface area	B	II-III
Risk score models Should be adapted by introducing a 1.5 multiplication factor for patients with either >10% body surface area involvement or those who are candidates for systemic or phototherapy	C	II-III
Risk management Should be carried out according to national guidelines and performed by either primary care physician or dermatologist. Target blood pressure and lipid levels are based on risk as previously calculated Antihypertensives and statins may be used as in general population	C	III

## Psoriasis and Microvascular Dysfunction

Beyond the well-documented association between psoriasis and CVD, current hypothesis placed the vascular network at the center of this circuit, describing a crucial role of the endothelium in the insurgence of psoriatic-dependent CVD. Indeed, it has been proposed that the cutaneous psoriatic lesion might induce a systemic inflammation through the immune-mediated activation of the endothelial compartment (56). In turn, this endothelial dysfunction, insurged at distant sites, could participate and concur to the development of cardiovascular pathological events (57).

Interestingly, psoriasis has been showed to impact early on the vascular compartment, even before the development of CV diseases, causing subclinical alterations typical of CVDs. Ludwig et al. used computed tomography to quantify the coronary artery calcification (CAC) as a biomarker of coronary atherosclerosis and a predictor of future cardiovascular events in patients with psoriasis and controls (58). An increased prevalence (59.4% vs 28.1%, *p*=0.015) and severity (3.7 vs 0.0, *p*=0.019) of CAC, assessed with Agatston score, has been demonstrated in psoriatic patients with a negative history of current or previous heart problems. In the same study, psoriasis has been pointed as an independent risk factor for CAC after multiple linear regression calculations. Multiple studies highlighted the presence of microvascular dysfunction, one of the earliest signs of CVDs, in psoriatic patients. Coronary microvascular dysfunction indeed indicates an abnormal regulation of the coronary microcirculation resulting in reduced myocardial blood flow in the absence of epicardial coronary arteries stenosis. The coronary flow reserve (CFR) analysis detects the damage to the microvascular heart circulation. CFR is the capacity of the coronary circulation to dilate and therefore to enhance its flow following an increased metabolic demand and it is related to the microvascular function as the 90% of the resistances of the coronary circulation are localized in the endocardial small vessels. The ratio between the maximal possible and the resting blood flow in normal subjects is >2.5. Intriguingly, we recently evaluated the CFR by transthoracic echocardiography inducing the hyperaemic stimulus with adenosine, showing a reduction of CFR in patients with psoriasis compared with healthy controls (*p*=0.02) (59). The risk of an abnormal CFR was higher in patients with a greater degree of psoriasis severity and this association was independent of traditional cardiovascular risk factors (60). Arterial stiffness, a surrogate of endothelial dysfunction and predictor of CV risk, was measured by Gisondi et al. in patients with moderate-severe psoriasis, by determining carotid-femoral and carotid-radial pulse wave velocity (PWVcf, PWVcr) (61). These patients, as compared to controls, showed a significantly increased PWVcf, even after adjustment for age, gender, smoking, hypertension and body mass index (8.78 ± 1.98 vs 7.78 ± 2.0 m/s; *p*=0.03). Moreover, a direct correlation between PWVcf and psoriasis duration (years) was evidenced (r=0.58; *p*=0.0001).

Taken together, different methodical approaches suggest an association between psoriasis and the microvascular dysfunction. Intriguingly, the immune-mediated dysfunction of endothelial cells in the vasculature is profoundly associated to the pathogenesis of several cardiovascular disorders. Indeed, the affected coronary arteries’ ability to increase coronary blood flow (vasodilatory abnormality) and/or the impaired coronary blood flow (coronary microvascular spasm) could insurge in severe psoriatic patients (62). Thus, data provide proof that psoriasis could be considered as an independent CV risk factor.

## Putative Inflammatory Circulating Factors Inducing Microvascular Dysfunction in Psoriasis

The immune-mediated endothelial activation is a process in which pro-inflammatory cytokines, integrins and multiple analytes contribute to trigger and propagate inflammation (63). A prolonged immune stimulation and an excessive or unbalanced production of inflammatory mediators might dysregulate this finely-tuned process.

Notably, the pathogenesis of psoriasis is considerably mediated by T helper (Th) type 1 activation and Th17 immune responses and the consequent overproduction of cytokines (e.g., Tumor necrosis factor-α TNF-α, IL-23, and IL-17) that ultimately generate a systemic proinflammatory environment. In this context, the chronic exposure of multiple circulating factors might play a key role in the endothelial priming and microvascular dysfunction at distant sites.

**Figure 1:**
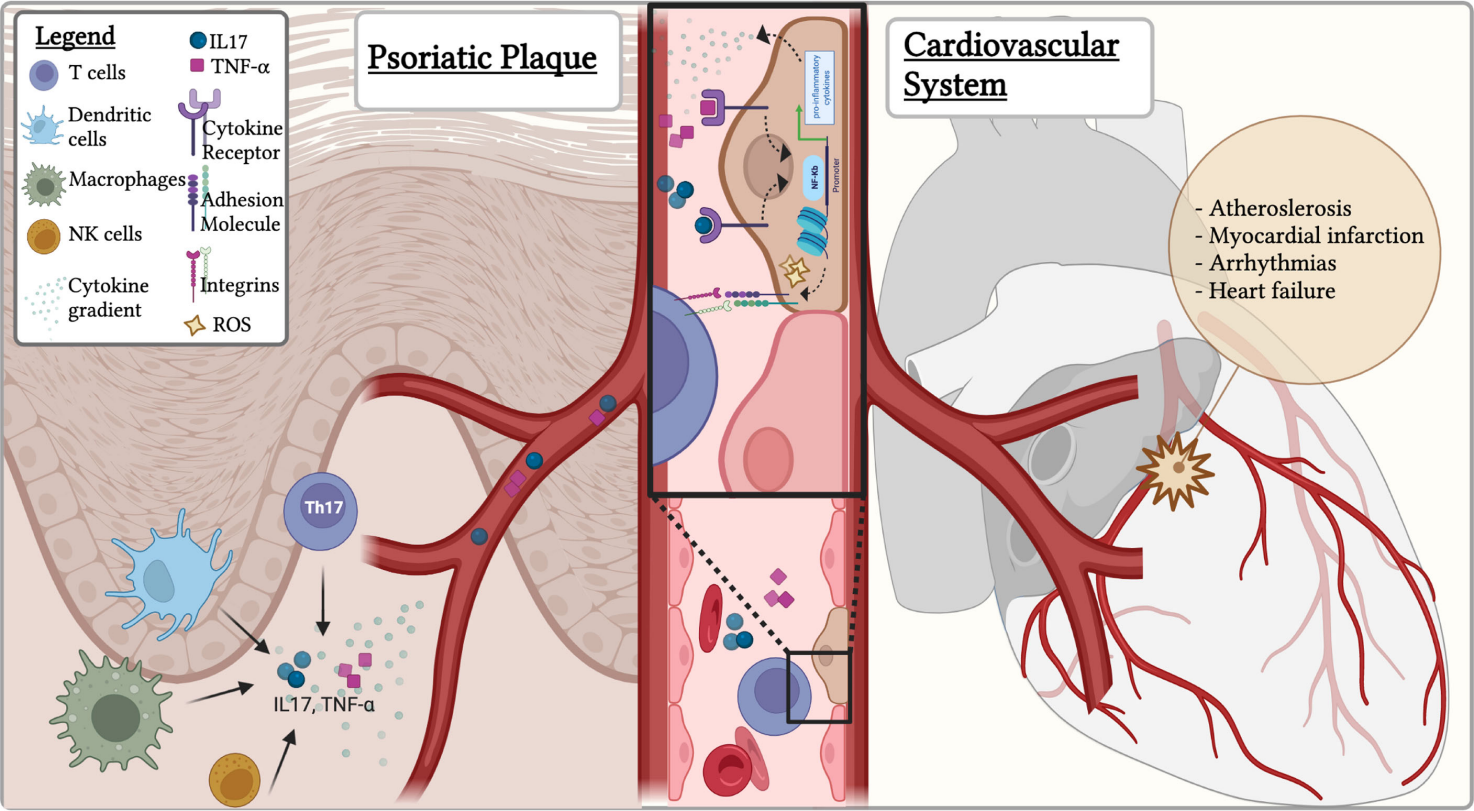
The immune-mediated pathogenetic link between psoriasis and cardiovascular disorders: triggering the endothelial dysfunction. In the psoriatic plaque, immune cells, including Th17 promote the generation and release of circulating inflammatory mediators as IL-17 and TNF-α. TNF-α caused NF-κB activation, subsequently leading to increased expression of multiple pro-inflammatory cytokines in the psoriatic skin. In endothelial cells, IL-17A receptor drives the production of TNF-α, IL-1β, CCL2, and the expression of ICAM- 1 leading to the endothelial dysfunction involved in the pathophysiology of multiple immune-mediated CVDs. The IL-17 also triggers the accumulation of ROS, in particular $O_{2}^{-}$ within the endothelium further contributing to endothelial dysfunction and CVD progression.

Endothelial dysfunction is referred to the imbalance between the release of vasoprotective/vasorelaxant mediators and pathological vasoconstricting substances. Despite the precise mechanisms mediating these vascular impairments are unclear, putative circulating analytes mediating the psoriatic immune-mediated endothelial dysfunction have been proposed. Among them, IL-17, TNF-α, reactive oxygen species (ROS), IFN-γ and Angiotensin-converting Enzyme, Renin and Endothelin-1(ET1) seem to play a crucial role (64–67) (**Figure 1**).

IL-17 is a pro-inflammatory cytokine formerly thought to be generated in the psoriatic plaque only by a subset of CD4+ T cells (Th17), concomitantly with IL-6, IL-21, IL-22 and TNF-α. Nowadays, also dendritic cells, natural killer cells, macrophages, and γδ-T cells have been recognized as important players in the IL-17 generation (68). A variety of mechanisms, including exogenous and inflammatory stimuli, might trigger the initial activation of Th17 cells, leading to the strong IL-17 release within the skin. Notably, the IL-17 receptor is ubiquitously expressed in endothelial cells of the vascular network. Here, once activated, IL-17 receptor drives the production of TNF-α, IL-1β, CCL2, and the expression of adhesion molecules as intercellular adhesion molecule 1 (ICAM-1). The upregulation of chemokines and adhesion molecules is associated to the endothelial activation, a process that has been recently linked to the endothelial dysfunction featuring the pathophysiology of multiple immune-mediated CVDs (67). To note, IL-17 might be responsible for an additional determinant of the endothelial dysfunction in the setting of vascular disease: the massive accumulation of reactive oxygen species, in particular of the vascular superoxide ( $O_{2}^{-}$ ) within the endothelium. In pre-clinical atherosclerosis model induced by a high-fat-diet-feeding, it has been reported that IL-17 caused the increased production of $O_{2}^{-}$ superoxide in the aortic vessel (69). Nonetheless, precise mechanisms by which IL-17, induces vascular $O_{2}^{-}$ production remain unclear. Mounting evidence reveals that IL-17 accumulation might underpin i) NADPH oxidase activation in vascular smooth muscle cells, and ii) superoxide production *via* recruiting inflammatory cells (70) (**Figure 1**). Overall, the oxidative stress has a key role in the development of CVD in patients with psoriasis. Indeed, CVDs are generally characterized by an imbalance between ROS generation and degradation made by antioxidants or ROS-catalysing enzymes, finally leading to a significant deviation from the homeostatic state (71).

Together with IL-17, TNF-α contributes to the pathogenesis of psoriasis. TNF-α is a multifunctional cytokine that play a fundamental role in mediating inflammation, immune responses, and apoptosis. Notably, higher levels of TNF-α were found in skin lesions of psoriatic patients (72, 73). More, locally produced TNF-α caused NF-κB activation in psoriatic lesions, subsequently leading to increased expression of multiple pro-inflammatory cytokines; possibly concurring to the endothelial activation, and eventually dysfunction, at distant sites (**Figure 1**). Moreover, TNF supports the proinflammatory effects of IL-17 (74). The two cytokines indeed synergistically promote the release of key molecules for psoriasis pathogenesis, such as β-defensin 4 and S100A7 (75).

Accordingly, these results support the assumption that a local induction of multiple inflammatory mediators in psoriasis plaques determines an effect in cells outside the skin, such as endothelial cells, altering the vascular function and eventually leading to CVDs. Unquestionably, a deep investigation of the cellular and molecular interplay involved in the skin/endothelium/cardiovascular system crosstalk will be crucial for further characterize the pathophysiological link between psoriasis and CVDs. For instance, extracellular vesicles-dependent mechanisms could be an interesting target that still remains to be addressed. Accordingly, future studies aimed at investigating this possibility might also pave the way for innovative therapeutic strategies.

## Targeting Key Inflammatory Pathways to Reduce CV Risk in Psoriatic Patients

Beside the evidence of the psoriasis-CVD crosstalk, several efforts have been made to understand whether a systemic treatment of psoriasis may lead to the control of the systemic inflammation associated with the skin manifestations, and to limit the risk of CV events (55, 76–78). The 2021 Group for Research and Assessment of Psoriasis and Psoriatic arthritis (GRAPPA) treatment recommendations for psoriasis are summarized in (79). Interestingly, EULAR guidelines report that disease activity should be controlled to lower CVD risk in all patients with rheumatoid arthritis, ankylosing spondylitis or psoriatic arthritis (80).

Classical disease-modifying antirheumatic drugs (DMARDs) routinely used in psoriasis include methotrexate, cyclosporine and acitretin. Among them, methotrexate (MTX) has been shown to be superior not only to topical treatments and phototherapy, but also to cyclosporine and acitretin in reducing the risk of MACE in patients with psoriasis (77, 81). In 2005, Prodanowich et al. firstly demonstrated the protective role of MTX on vascular disease in patients with psoriasis (82). In line with this, MTX has been associated with 21% lower risk for CVD and a 18% lower risk for MI in a systematic review and meta-analysis of 10 observational studies in which MTX was administered in patients with psoriasis, rheumatoid arthritis or polyarthritis (83). On the other hand, the role of cyclosporin and acitretin is still considered controversial. Indeed, they are associated with side effects such as renal failure, hypertension and dyslipidemia that can negatively impact on CV risk (84).

In the recent years, the attention has been focused on biological drugs as they target key inflammatory molecules involved in the pathogenesis of the disease, allowing a complete clearance of the skin lesion in almost all patients and a long-term control of the condition (6).

TNFα inhibitors commonly used in psoriatic disease include infliximab, etanercept, adalimumab and certolizumab. Patients treated with anti TNF-α for over 24 weeks demonstrated a reduction in inflammation markers related with endothelial dysfunction, such as CRP e vascular endothelial growth factor (VEGF) (85). These data have been confirmed in a randomized controlled trial reporting a reduction in inflammatory CVD markers such as GlycA, IL6, CRP, and TNF-α (86). Consistently, it has been noted a reduction in vascular inflammation assessed by the decreased signal intensity on FDG PET/CT in 115 patients after one year of treatment. Interestingly, a reduction of 75% in skin diseases severity was associated with a greater improvement in aortic vascular inflammation (87). The relevant role of TNF-α inhibitors in modulating the CV risk in psoriasis was also demonstrated by their ability to reverse the microvascular dysfunction measured by CFR. Indeed, a prospective study evaluating CFR in 37 consecutive patients with moderate to severe psoriasis before and after 6 months of treatment with TNF-α inhibitors reported a CFR increase from 2.2 ± 0.7 to 3.02 ± 0.8 (*p*<0.0001) (59). Other studies evaluated the impact of TNF-α inhibitors in psoriasis on several different CV imaging biomarkers. In particular, TNF-α inhibitors demonstrated a favourable impact on the arterial stiffness, assessed by the gold standard aortic pulse wave velocity (aPWV) (88), and decreased burden of noncalcified coronary plaques (89). Moreover, subclinical left and right ventricular myocardial dysfunctions have demonstrated to be ameliorated following anti TNF-α therapy in patients with severe psoriasis (90, 91). Although, to date, randomized placebo-controlled trials (RCT) evaluating the impact of biologics on CV risk in psoriasis are lacking, a systemic meta-analysis revealed that TNF-α inhibitors were related with fewer cardiovascular events compared to topical treatment/phototherapy (RR 0.58) or methotrexate (RR 0.67) (92). A retrospective cohort study demonstrated the beneficial effect of TNF-α inhibitors on myocardial protection by reducing the risk of MI compared with topical agents (adjusted hazard ratio, 0.50; 95% CI, 0.32-0.79) (93). The same result was reached by a large-scale observational study that confirmed a reduction by 11.2% of CV event risk in psoriasis patients exposed to TNF-α inhibitors for 6 months compared to those who received phototherapy (94). Similarly, in another study, TNF-α inhibitors have proved to be superior to methotrexate in reducing the risk of MACE (95).

Recently developed IL-17 inhibitors include the anti-IL-17 antibodies secukinumab and ixekizumab and the anti-IL-17 receptor antibody brodalumab. Preclinical studies have shown that IL-17 inhibitors diminished peripheral oxidative stress levels, proinflammatory cytokines, and vascular inflammation in psoriasis (96). In a prospective, observational study including 215 patients treated with different biologic therapies and completing one year of follow up, patients undergoing IL-17 inhibitors, along with the reduction of CRP and HDL serum levels, showed the greatest reduction in coronary plaque indices assessed by coronary computed tomography angiography as compared to groups treated with other biologics (89). The CARIMA study found an improvement of the endothelial function assessed by a reversal of baseline flow-mediated dilation, in psoriasis patients treated with secukinumab, revealing its beneficial effect on CV risk (97). On the contrary, another paper showed no effects on aortic vascular inflammation and cardiometabolic biomarkers in moderate-severe psoriasis patients treated with secukinumab compared to placebo (98).

The anti- IL-12/IL-23 antibody ustekinumab and the anti-IL-23/IL-39 antibodies guselkumab, risankizumab and tildrakizumab are currently approved for the treatment of psoriasis. In several studies, ustekinumab has demonstrated to have a neutral impact on MACE (99, 100). A recent meta-analysis of RCT, attempting to explore the impact of biologics on serologic and imaging biomarkers of CV risk, pointed out that adalimumab and secukinumab did not cause a significant improvement in imaging markers. Conversely, ustekinumab treatment promoted the reduction of aortic vascular inflammation at week 12, albeit this was not confirmed at week 52. Among all biologics, adalimumab was the most efficient in inducing a reduction of the serum markers CRP, TNF-α, IL-6, and GlycA (101).

Overall, these data point toward the improvement of imaging and serum biomarkers for CVD risk in patients with psoriasis treated with biologics targeting TNF-α, IL-23 and IL-17; moreover, therapies with biologics, by controlling remote skin inflammation, may have the potential to prevent the development of CVDs.

## Conclusions

The association between psoriasis and CVD is promoted by the coexistence of traditional modifiable CV risk factors (hypertension, diabetes, hyperlipidemia, obesity, and metabolic syndrome) and chronic systemic inflammation. The latter might directly impinge on the vascular compartment leading to a pan-arterial inflammation and, in particular, to a microvascular dysfunction. In this context, biological drugs, targeting psoriasis key inflammatory molecules, demonstrated a positive outcome on both skin manifestations and cardiovascular involvement. However, further clinical studies are needed to investigate the potential beneficial effects of biologic agents in the reduction of CV risk in psoriatic patients.

## Author Contributions

All authors listed have made a substantial, direct, and intellectual contribution to the work, and approved it for publication.

## Funding

BM and RA received individual research grants from Istituto di Ricerca Pediatrica Fondazione Città della Speranza.

## Conflict of Interest

The authors declare that the research was conducted in the absence of any commercial or financial relationships that could be construed as a potential conflict of interest.

## Publisher’s Note

All claims expressed in this article are solely those of the authors and do not necessarily represent those of their affiliated organizations, or those of the publisher, the editors and the reviewers. Any product that may be evaluated in this article, or claim that may be made by its manufacturer, is not guaranteed or endorsed by the publisher.
